# Optimizing cooperation between general practitioners, occupational health and rehabilitation physicians in Germany: a qualitative study

**DOI:** 10.1007/s00420-017-1239-6

**Published:** 2017-07-05

**Authors:** Jan Stratil, Monika A. Rieger, Susanne Voelter-Mahlknecht

**Affiliations:** Institute of Occupational and Social Medicine and Health Services Research (Institut fuer Arbeitsmedizin, Sozialmedizin und Versorgungsforschung), University Hospital Tübingen, Wilhelmstrasse 27, 72074 Tübingen, Germany

**Keywords:** General practice, Occupational medicine, Rehabilitation, Health services research, Interfaces, Interprofessional cooperation

## Abstract

**Purpose:**

To achieve successful medical rehabilitation and timely return to work, general practitioners, occupational health and rehabilitation physicians need to cooperate effectively. This cooperation, however, can be hampered by organizational, interpersonal, and structural barriers. In this article, we present and discuss suggestions proposed by physicians and patients on how these barriers can be overcome.

**Methods:**

We conducted eight qualitative focus group discussions with general practitioners (GPs), occupational health physicians (OPs), rehabilitation physicians (RPs) and rehabilitation patients, which we analyzed with qualitative content analysis methods.

**Results:**

Room for improvement exists with regard to (1) regulation (e.g. formalized role and obligatory input of occupational physicians), (2) finance (e.g. financial incentives for physicians based on the quality of the application), (3) technology (e.g. communication by email), (4) organizational procedures (e.g. provision of workplace descriptions to RPs on a routine basis), (5) education and information (e.g. joint educational programs, measures to improve the image of OPs), and (6) promotion of cooperation (e.g. between OPs and GPs in regards to the application process).

**Conclusions:**

Many suggestions are practical and could be implemented into the daily routine of physicians, while others demand multi-level, multi-stakeholder approaches. Our findings are supported by numerous international studies (especially from Western Europe). Future quantitative research could assess the relative weight of these findings. Feasibility and effectiveness of the proposed suggestions should be tested in controlled interventional studies.

**Electronic supplementary material:**

The online version of this article (doi:10.1007/s00420-017-1239-6) contains supplementary material, which is available to authorized users.

## Background

Occupational health physicians (OPs), general practitioners (GPs), and rehabilitation physicians (RPs) fulfill different functions in the rehabilitation process, which need to be connected and coordinated effectively to achieve successful medical and occupational rehabilitation in employees.

International studies show that representatives of all three physician groups agree that their cooperation and communication are necessary for successful rehabilitation (van Amstel and Buijs 2000; Friesen et al. 2001; Edlund and Dahlgren 2002; Schochat et al. 2003; Beaumont 2003a; Rijkenberg 2012).

These statements are supported by several studies from Germany, which have indicated that improved cooperation in the rehabilitation process and especially the inclusion of OPs is beneficial in improving the occupational participation of patients (Kuehn et al. 2008; Mueller et al. 2009; Schwarze et al. 2013; Bethge 2016).

Moreover, a number of interventions have been found to improve the work-related health of patients in international literature reviews (e.g. in regards to reduced sick leave and time to first return to work). A number of these interventions lie within the responsibility of OPs in the German health care system, including rehabilitation treatment tailored to demands of the patient’s workplace, work accommodations such as ergonomic improvements, and early contact of the worker to the workplace (Franche et al. 2005; MacEachen et al. 2006; Carroll et al. 2010; van Vilsteren et al. 2015).

Insufficient cooperation and communication between GPs, OPs and RPs has been identified as a relevant problem in numerous studies, and the need for improvements is acknowledged by all those involved (Seidel et al. 2003; Beaumont 2003a, b; Beach and Watt 2003; Vroeijenstijn-Nguyen and Brenner 2007; Mueller et al. 2013; Rijkenberg et al. 2013). In particular, several studies have identified an insufficient flow of information from and to OPs as a main barrier to a streamlined rehabilitation process in employees (Schupp 2001; Dasinger et al. 2001).

Surveys conducted among RPs, OPs, and rehabilitation patients from Austria, the Netherlands, and Belgium found a low intensity of communication and cooperation between OPs and RPs in all three countries (van Amstel and Buijs 2000; Vroeijenstijn-Nguyen and Brenner 2007; Rijkenberg 2012). German surveys in particular reported strong feelings of being excluded from the rehabilitation process among OPs (Seidel et al. 2003; Tavs 2005; Luedemann 2006; Mueller et al. 2013). These findings have been underlined by several German studies which reported that communication between RPs and OPs still does not take place on a regular basis (Schwarze et al. 2013) and that OPs were often informed about their patient’s rehabilitation treatment months after their discharge, if at all (Behrens 2000; Manecke et al. 2008). Another study conducted in Germany found that OPs were mentioned or addressed in less than 1/8 of all discharge letters from rehabilitation clinics, and most of these references to OPs were negative (Jankowiak et al. 2013). These findings have been confirmed by two recent international literature reviews on the cooperation between OPs and RPs (Rijkenberg et al. 2013; Voelter-Mahlknecht and Rieger 2014).

Similar findings have been reported in a literature review on the cooperation between GPs and OPs (Mosshammer et al. 2011). The rehabilitation process has been identified as a main interface between GPs and OPs (Mosshammer et al. 2011), and room and need for improvement at this interface has been identified by numerous studies, including several recent studies from Germany (Beaumont 2003a; Beach and Watt 2003; Mosshammer et al. 2011, 2012, 2016).

In a previous publication within the same qualitative research project, we identified organizational, interpersonal, and structural barriers leading to low levels of cooperation (Stratil et al. 2017). Organizational barriers included: Missing contact details of OPs, low reachability of RPs, OPs, and GPs, time restrictions of RPs and GPs, and problems caused by the RPs’ need for fast coordination with OPs on short notice. In regards to interpersonal barriers, patients as well as physicians reported that low levels of trust and poor relations between employees and OPs might be a barrier, as patients have to agree to OPs being included in the rehabilitation process and being provided with patient data. Furthermore, patients and physicians have expressed concerns that OPs might not follow confidentiality regulations and might have conflicts of interest due to their relationship with the employer. Other interpersonal barriers included lacking initiative or interest in communication by RPs, OPs, or GPs and a low perceived need to cooperate with OPs. For example, while OPs perceived a third-party workplace description as important for successful rehabilitations, RPs felt they were able to sufficiently assess the patients’ workplaces, believed the integration of OPs into the occupational reintegration process as rarely necessary, and considered OPs to be optional recipients of the rehabilitation report (Stratil et al. 2017). These findings are in line with other studies conducted in Germany or Western Europe (Valk and van den Broek-Porius 2007; Vroeijenstijn-Nguyen and Brenner 2007; Mosshammer et al. 2011; Rijkenberg 2012; Mosshammer et al. 2014).

The overall aim of this research project was to identify barriers to and ways to improve the cooperation between RPs, OPs, and GPs in the rehabilitation process in Germany (Voelter-Mahlknecht et al. 2017). Following recommendations of the Medical Research Council (MRC) (Campbell et al. 2000) we first conducted a systematic literature review (Voelter-Mahlknecht and Rieger 2014). The first part of the qualitative study focused on the assessment of the cooperation as well as the identification of barriers and determinants for good cooperation and communication (Stratil et al. 2017). The present, second part focuses on possible solutions as reported by the four groups of stakeholders. In particular, we aim to answer the following questions:

1. What kind of practical advice for improved communication and cooperation at the interface between GPs, OPs and RPs can be deduced from the personal experiences of the different stakeholders?
2. What opportunities for optimization beyond the improvement of communication and cooperation do the medical parties and the rehabilitation patients point out?

Based on our review of the literature, we will focus on the role of OPs in the rehabilitation process (Rijkenberg et al. 2013; Voelter-Mahlknecht and Rieger 2014).

## Methods

This explorative qualitative study is based on eight Focus Group Discussions (FGDs) and used qualitative content analysis for data analysis (Mayring 2014). These methods are laid out in more detail in our study protocol and our publication on barriers to cooperation, in which we also contextualize the roles of the protagonists as well as the specific barriers addressed within the German health care system (Voelter-Mahlknecht et al. 2017; Stratil et al. 2017). In our reporting we followed recommendations outlined in the COREQ statement (Tong et al. 2007).

In short, two FGDs with seven participants on average (ranging from 4 to 10) were conducted with each of the three professional groups (GPs, RPs and OPs) as well as with patients as a fourth stakeholder group. The semi-structured FGDs with a duration between 85 and 99 min were conducted between February and May 2015. They were conducted by one of two female researchers of which one is an associate professor for occupational, social and environmental medicine for social medicine (author SVM) and the other an occupational safety engineer. Both were working for the Institute of Occupational and Social Medicine and Health Services Research at the University Hospital Tübingen, had previous experience in qualitative research and received theoretical training in our institute.

We informed the participants prior to the FGD about the professional background of the interviewer and the aim of the research project. One interviewer (SVM) was already acquainted with three OPs and one GP.

The study’s purposive sample is shown in Table 1. We used a purposive sampling technique aiming for maximal structural variation in the composition of our sample in order to represent the heterogeneity of the stakeholder involved in the process we aimed for (Palinkas et al. 2015). OPs were recruited via telephone from members of the Association of German Occupational and Company Physicians (Verband Deutscher Betriebs- und Werksärzte (VDBW)) by one of the authors (JMS). RPs and patients were recruited through cooperation with the rehabilitation clinics Treatment Center Federsee (Therapiezentrum Federsee) in Bad Buchau (specializing in orthopedics, oncology and rheumatology) and the Huettenbuehl clinic of the Rehabilitation Center Bad Duerrheim (Reha-Zentrum Bad Duerrheim, Klinik Huettenbuehl) in Bad Duerrheim (specializing in psychosomatic and mental health) via contact persons. GPs were recruited via email from medical practices associated with the Department for General Medicine at the University of Tübingen.

**Table 1:** Characteristics of the study population

Physicians	General practitioners	Occupational health physicians	Rehabilitation physicians	Patients	Patients
Participants	*n* = 22	*n* = 9	*n* = 12	*n* = 15	Participants
Age average [median/(range)]	57/(40–67) years	55/(45–65) years	48/(34–58) years	53/(22–63) years	Age [median/(range)]
Gender: N. female	*n* = 9	*n* = 5	*n* = 6	*n* = 8	Gender: N. female
Work experience as physician	27/(13–40) years	29/(12–39) years	13/(6–30) years	One: *n* = 4 Two: *n* = 1 Three: *n* = 1	Previous rehabilitation therapies
Work experience in specialization [median/(range)]	21/(7–33) years	20/(1–32) years	11/(3–31) years		
Type of employment	Solo practice: *n* = 13 Group practice: *n* = 9	Employed at enterprise: *n* = 1 Employed in occupational health service: *n* = 4 Freelance: *n* = 4		21 days: *n* = 4 28 days: *n* = 3 35 days: *n* = 5 >35 days: *n* = 3	Planned duration of rehabilitation (days)
Practice site	Urban: *n* = 2 Rural: *n* = 10 Mixed: *n* = 10	Urban: *n* = 5 Rural: *n* = 0 Mixed: *n* = 4		Mental health *n* = 5 Musculoskeletal *n* = 5	Reason for rehabilitation
Practice size (patients per 3 months)	<700: *n* = 2 700–1400: *n* = 14 >1400: *n* = 5	Responsible for SME: *n* = 8		Office work: *n* = 5 Industrial production: *n* = 3 Construction work: *n* = 1 Logistic sector: *n* = 1 Nursing care: *n* = 2 Pedagogue: *n* = 1 Cleaner: *n* = 1	Occupation
Rehabilitation applications [median/(range)]	35/(5–50) per year				
Within catchment area of a company medical service?	In town: *n* = 7 In the country: *n* = 3 Both: *n* = 2 Without: *n* = 8			Small or medium enterprises: *n* = 7	Type of employer
				Business has OP: *n* = 8 Patient knows OP: *n* = 7	Relationship to OP (responses by patients)
Setting of data collection	Meeting room in the University Hospital Tübingen resp. in our institute in Tübingen	Meeting room in our institute in Tübingen and conference room in Stuttgart	Meeting room in rehabilitation clinics	Meeting room in rehabilitation clinics	

The pilot tested FGD-guide focused on (1) attitudes towards rehabilitation therapy (warm-up question), (2) the perceived role and function of OPs, GPs, and RPs in the rehabilitation process, (3) the informational need of patients and medical stakeholders, and (4) the perceived quality and intensity of cooperation and communication at the interfaces between the different groups. The full interview guide can be provided upon request.

The discussions were digitally recorded on audio and video, with the videos being deleted after the pseudonymization of the transcript. No field notes were taken. The transcribed and pseudonymized interviews were assessed with the methodological orientation of content analysis using the method of qualitative content analysis (Mayring 2014) and the software MAXQDA 11 (VERBI GmbH, Berlin, Germany). Two to three researchers went through the transcripts line by line and built inductive categories from the material. After coding three out of the eight transcripts we could identify no further categories and therefore assumed saturation. We then revised the coding frame and assessed whether it met the research questions. Next, we applied the categories deductively on the complete set of all eight transcripts. In order to control for subjective blurring and to achieve intersubjective creditability, two to three persons created and applies the categories partly independently from each other, and partly in close discussion (Mayring 2014). The transcripts were not returned to participants for comments or corrections, but in January 2015 we conducted a workshop for content validation in which we presented a summary of the content of the FGDs and preliminary findings to the participants. Representatives of all parties were invited and a total of 16 GPs and OPs participated.

## Results

We identified four main categories: (I) “perceived interfaces between the protagonists”, (II) “perceived problems in the rehabilitation process”, (III) “perceptions of and attitudes towards the own group and other stakeholders” and (IV) “perceived role of protagonists in the rehabilitation process”. The category system is displayed in Electronic Supplementary Material, Annex I. The first two categories were the focus of the preceding publication on barriers to communication and cooperation. This publication is primarily based on the categories “Suggestions for improvements” which is a subcategory to several of the categories (i.e; II.a–g; III, IV) in the category system.

We assigned the suggestions proposed by our four groups of stakeholders in six categories: (O) organizational, (T) technology, (F) finance, (R) regulation, (E) educational and information, and (P) promotion of cooperation. These categories are displayed in Table 2 with exemplary text passages and suggestions in Table 3.

**Table 2:** Coding frame of categories included in this study, and coding examples

**Suggestions related to**
**Regulation**
**M1**: “…everywhere where there’s an interface with the workplace, that’s where we [OPs] play an important role. I don’t understand why it’s not standard for us to be the actors during the progressive reintegration phase. It should be like that as a matter of principle, but it’s not.” OP II, 150
**Financing**
**Interviewer:** “Missing diagnoses. Would it be possible to make it more attractive to GPs by increasing the remuneration for a rehabilitation application?” **M1:** “Absolutely.” **F2:** “I agree.”**M1:** “Absolutely.” RP II, 335–338
**Technical and technological solutions**
**F4:** “I can also imagine that calls are considered bothersome by the GP. If we could write an email now,… I believe that would be more helpful, if they could chose the time when to read this information themselves, or so.” RP I, 178–280
**Organizational procedures**
**M1:** “What you could do [to provide the occupational physician information if the patient doesn’t fully trust him/her], would be to simply reduce it to the sociomedical assessment. So that he [the OP] doesn’t get all of the other information, just the sociomedical assessment.” RP I, 121–125
**Education and Information**
**M2**: “…the company physicians are always rather exotic for the other two groups, doing something that a general practitioner doesn’t really know about, and the same for the reha-physician. And this lack of knowledge about each other naturally leads to misjudgments.” OP II, 101–102
**Promoting cooperation**
M3: “… it would naturally be nice if, when you work in a company, and you always had similar or the same rehabilitation clinics where you sent people. Then contact could gradually be built up.” OP II, 254–257

In brackets: section in the MAXQDA file, in bold: pseudonymization codes of the interview partners

*F* female participant, *M* male participant, *OP* FGD with occupational health physician, *RP* FGD with rehabilitation physicians, *GP* FGD with general practitioners

**Table 3:** Suggestions for improving cooperation and their presumed acceptability, feasibility and efficacy, based on statements made by the participants in our interviews

	Main addressee	Intervention		Opinion in interviews			
				GPs	OPs	RPs	Rehab
Application	OPs/GPs	P.1 P.2	Promote cooperation between OPs and GPs on application: OPs could add assessment to GPs application OPs could write objection to rejected applications	↑	↑	U	U
		R.1 R.2	Funding agencies should make an OP’s contribution to application form (i.e. statement (R.1)/work place description (R.2)) an obligatory perquisite for acceptance of application	↓	↑	U	M
		F.1	Remuneration of doctors for filing rehabilitation applications should be increased	↑	↑	↑	U
		F.2	A conditional financial incentive for application forms containing all necessary information should be introduced	U	U	↑	U
Rehabilitation report	Funding agencies	R.3	Funding agencies should introduce regulations in order to shorten the rehabilitation report	↑	↑	M	U
		R.4	Funding agencies should introduce regulations to allow a division of the rehabilitation report into segments and have recombined and tailored reports send to recipients (i.e. OPs)	U	U	↑	U
		R.5	OPs should be obligatory recipients of the rehabilitation report	↓	↑	M	M
		R.6	The default status of OPs as recipient should be introduced, instead of an explicit opt-in decision of patients/RPs	U	↑	U	U
	OPs/RPs/GPs	O.5	OPs, RPs, and GPs should developing a joint definition or understanding of terms, i.e. regarding the patient’s ability to work	U	↑	U	U
	Funding agencies	T.1	A revised discharge letter with predefined terms relating to the patient’s ability to work should be introduced (i.e. by the DRV)	U	↑	U	U
Evaluation	Funding agencies	R.7	To improve evaluation of the rehabilitation, a structured follow-up program including medical consultation and examination i.e. by a GP should be introduced	↑	↑	U	U
		R.8	A structured post-discharge check-up conducted by OPs should by introduced (i.e. by the funding agencies)	↑	U	U	U
	RPs	O.2	Introduce an evaluation system based on rehabilitation clinics sending questionnaires to GPs 6 months after rehabilitation	↑	U	↑	U
		O.3	Have rehabilitation institutions send a reminder to GPs to evaluate the results of rehabilitation	↑	U	↑	U
Occupational reintegration	RPs/OPs	P.3	Promote RPs reaching out to OPs if continued employment of patient is at risk	U	↑	U	U
	Funding agencies	R.9	Have OPs contribution to occupational reintegration made obligatory (i.e. by the funding agency)	↓	M	↓	M
	Employer/OPs	O.4	OPs could make an arrangement with the employer, to have the employers’ acceptance of the RPs’ proposal for occupational reintegration to depend on the OPs assessment	U	↑	U	U
Post-rehab. treatment	Funding agencies	F.3	Organize financing of post-rehabilitation treatment through the rehabilitation institutions (i.e. through voucher booklets)	↑	U	U	U
Communication	OPs, RPs, GPs	T.2	Increase the use of e-mails in the communication between OPs, GPs, and RPs (i.e. by introducing appropriate software)	↑	↑	↑	U
Joint medical education	OPs, RPs, GPs	E.2	Introduce/increase joint continuing medical education programs between RPs, OPs, and GPs	M	↑	↑	U
	OPs	E.3	Introduce education programs within companies to provide RPs and GPs insight into occupational health aspects	U	↑	↑	U
OP–RP-communication	OPs	P.4	Establishing lasting cooperation between OPs/employers and selected rehabilitation institutions	U	↑	U	U
	Employers	O.1	Have HR departments send the OP contact details or work place description by default	U	↑	↑	U
	OPs	E.1	Encourage OPs to file applications more often to increase their visibility	U	↑	U	U
Cooperation with OPs in general	OPs	E.4	OPs should focus more on informing and educating GPs/RPs/patients better about OPs’ role and functions	U	↑	↑	↑
	Prof. organizations	E.5	Professional associations should focus on informing and educating GPs/RPs/patients better about OPs’ role and functions	U	↑	U	U

↓, rejected; ↑, supported/suggested; U, attitude unclear; M, mixed responses. Categories of the suggestions: E, Education and Information; R, Regulation; F, Financing; T, technical and technological salutations; P, promoting cooperation; interviewees: GP, general practitioners; OPs, occupational health physicians; RPs, rehabilitation physicians; Rehab, rehabilitation patients

### Suggestions to improve the interfaces prior to rehabilitation

In Germany, patients in need of rehabilitation therapy need to apply for funding at their insurance, in general the public German Pension Insurance (DRV). The application needs to include a health assessment report by a GP, OP, or another medical specialist. GPs in our interview criticized high rejection rates, a time consuming application process, insufficient remuneration for the medical support of the patients’ application, and lack of compensation for filing objections to rejected applications. Some OPs therefore suggested that an improved cooperation between GPs and OPs in the application process could be beneficial for GPs and patients. It was proposed, that (P.1) OPs could add an OP’s assessment to the GP’s application, which could increase the acceptance rate in the view of both GPs and OPs. They also suggested (P.2) that OPs could file objections to rejected applications, which would save time for GPs. GPs and OPs themselves could promote and initiate this form of cooperation with local and regional stakeholders, e.g. by promoting this form of cooperation by arguing with the mutual benefits of such arrangements.

### Interfaces at the beginning and during rehabilitation

RPs and OPs regarded the provision of information on the patient’s workplace and occupational setting as an important interface and reported missing contact details of OPs as a main barrier, although the application form calls for the OP’s contact details. Both groups of physicians agreed that the contact details of the patient’s OP should be provided to RPs by default. This could be accomplished by encouraging, incentivizing, or obliging patients and the physician supporting the application process to provide the OPs contact details to RPs. OPs suggested that (R.1) the funding agencies should make this segment an obligatory part of the application form, without which the application should be rejected. GPs rejected this proposal and suggested (F.1) a raise of the remuneration instead. An increase could motivate the physician to invest more time in this task and—as a result—improve the quality of the applications. RPs proposed (F.2) a conditional financial incentive for the physician for handing in an application fulfilling certain quality criteria (e.g. containing the OP’s contact details). Other suggestions made by OPs to improve the provision of RPs with an OP’s assessment of the workplace included (R.2) making such a description by the patient’s OP an obligatory part of the application form. This proposal was rejected by other OPs and the majority of RPs. As a less intrusive suggestion, one OP proposed (O.1) to have the companies’ HR-departments send (O.1a) the OP’s contact details or a workplace description (O.1b) to the OP to the patient’s rehabilitation clinics by default, as soon as this department was informed about the patient’s rehabilitation treatment. It was argued that this was only feasible in sufficiently large companies and RPs in both focus groups were hesitant about the provision of a workplace description by default, as they felt sufficiently able to assess the patient’s workplace in most cases and were concerned about being flooded with unnecessary information.

### Interfaces at the end of rehabilitation treatment

At the end of rehabilitation, RPs provide GPs and other physicians treating the rehabilitant with a short discharge letter, as well as with a more detailed and longer rehabilitation report, which should be send to the treating physicians within 14 days.

All participants advocated for improvements regarding this informational interface. (R.2) GPs, OPs, and RPs supported shortening the rehabilitation report, which would make changes in regards to the regulation of the funding agencies necessary. In their view this would reduce the time needed to write the report, lead to a faster delivery and could reduce information loss at the interface, as such as shorter reports are more likely to be read. Some RPs rejected this suggestion, as condensing the information might take even longer than writing a longer report and as the comprehensiveness of the report could be beneficial and timesaving for other physicians, as they could rely on the extensive medical history recorded in the rehabilitation clinic.

Some RPs suggested that (R.3) the rehabilitation report should be divided into sections, which could be combined based on the informational needs of the respective recipients. Such a letter would be shorter, have a higher information density and would more likely be read, and could overcome the barrier of patients’ and physicians’ concerns about data privacy, as no unnecessary personal information would be passed on. RPs stated that through a recombined rehabilitation report, it would be possible to pass on segments, e.g. relevant for occupational reintegration without providing personal or sensitive information (e.g. about the patient’s mental health), which otherwise could decrease the acceptability of providing the respective medical stakeholder with any information. OPs and GPs both stated they only needed parts of report in order to fulfill their function.

(R.4) Some OPs proposed that the rehabilitation report should be sent to OPs by default (R.5) or to change the default status of OPs as recipients of the rehabilitation report from an opt-in to and opt-out status: instead of actively adding the OP as a recipient, OPs should receive the rehabilitation report by default unless this is actively rejected by the patient. A number of GPs and RPs rejected these proposals. Some RPs even questioned whether OPs should receive the rehabilitation report at all. Even sending a shortened version of the report by default was rejected by some RPs.

### Interventions after the rehabilitation treatment

In the German health care system GPs work on a budget which limits the amount of diagnostic procedures and treatments that they can prescribe. GPs in our interviews argued that the treatment recommendations by the RPs posed a substantial financial burden on their budget and had led to conflicts with patients. GPs therefore suggested that the (F.3) post-rehabilitation treatment (e.g. physical therapy) should be paid via the rehabilitation institutions by their funding agencies, for example through a “voucher booklet” given to the patients by the rehabilitation clinic. Of note, consolidative programs following medical rehabilitation (i.e. functional trainings) are already reimbursed by the Pension Insurance. RPs acknowledged that financial and legal limitations constrained GPs during post-rehabilitation therapy, but argued that some of these issues could be solved through direct communication between GPs and RPs.

RPs, GPs, and OPs alike stated that an improved evaluation of the rehabilitation process would be desirable. Some RPs were more reserved, stating that an evaluation by GPs was not feasible due to time limitations and that the feedback would not have an impact, as this feedback would not change the working routine in the rehabilitation institutions. RPs suggested (O.3) introducing a feedback system based on questionnaires sent to patients or GPs by the rehabilitation clinic 6 months after discharge. Other GPs and RPs preferred a (R.6) structured follow-up program, which should include a medical consultation and examination by the GPs, rather than just a questionnaire survey. GPs pointed out that an evaluation system to be used by GPs already existed, but was rarely used in practice, i.e. due to time limitations and due to GPs forgetting about sending an evaluation. Participants in GP and RP FGDs suggested to have RPs (O.4) send the GPs a reminder at the respective deadline. GPs supported an active role of RPs in the evaluation process. OPs stated that they could play a role in the evaluation and feedback processes as well, e.g. (R.7) through a post-discharge check-ups.

OPs in our interviews suggested that (P.3) RPs should have to reach out to OPs in cases when the patient’s job was at risk, e.g. due to health-related restrictions which would affect the patient’s professional activities. RPs also should discuss possible consequences of the formal assessment of the patient’s ability to work could have for the patient (e.g. the risk of losing the job due to health restrictions). According to OPs, patients often were not aware of these consequences and these were not sufficiently addressed by the RPs.

A number of OPs argued that (R.8) including OPs in the return-to-work process should be made obligatory, which was disputed by other OPs. Another suggestion focused on strengthening the OPs’ role in the return-to-work process through the employer: (O.5) Employers could make the approval of the RPs’ proposals for a return-to-work dependent on the OPs’ decision. This would lead to RPs having to contact OPs on a regular basis. The OP who suggested this had such an arrangement with her employer, which led to her being involved in the occupational reintegration of her patients in nearly all instances.

A differing understanding of terms related to patient’s ability to work (e.g. the term *piecework*, or Akkordarbeit in German) was considered a problem by RPs and OPs. Some OPs suggested (O.6) introducing a common language by developing joint definitions, while another OP suggested (T.1) developing a discharge letter which included a predefined list of terms relating to the patients’ ability to work. By using this tool, RPs could clearly communicate their assessment on which tasks patients were no longer able to perform.

### Suggestions for improving the rehabilitation process in general

According to some OPs, OPs needed to increase their visibility in order to improve their integration into the rehabilitation process and to build up professional and personal relationships with RPs. To raise RPs’ level of trust and increase the OPs’ visibility, OPs suggested (P.4) establishing lasting cooperation between OPs or employers with selected rehabilitation clinics and to (E.1) encourage OPs to file applications for rehabilitation more often.

A number of OPs supported increasing contact and interaction of RPs and OPs, e.g. through (E.2) joint continuing medical education programs. The concept of joint educational programs was supported in both professional groups. Some OPs also suggested (E.3) education programs within companies to give RPs insight into patients’ work places. Some OPs believed that the lack of cooperation was caused by insufficient knowledge about the OPs’ role, capabilities, and code of confidentiality. According to OPs, educating RPs on the OP’s role and function in the rehabilitation process could overcome this issue. Some OPs proposed that they (E.4) should better explain their position and role to patients and other physicians, while others suggested that (E.5) professional associations should take a stand to strengthen the role of OPs.

(T.2) GPs, RPs, and OPs all supported to increase the use of emails to overcome the barrier of limited time and conflicting schedules through time lags caused by sending letters by post. RPs stated that data privacy regulations currently prohibit the use of email in rehabilitation clinics, but that this could be solved through the introduction of proper encryption software.

## Discussion

The participants in our study proposed suggestions on how problems in the rehabilitation process and barriers to cooperation between OPs, GPs, and RPs could be overcome. These suggestions referred to (1) regulation, (2) financing, (3) organizational procedures, (4) education and information, and (5) promotion of cooperation.

While some of the suggestions are rooted in problems specific to the German health care system, including some suggestions regarding financing or organizational procedures, and may be limited to the German setting or health care systems similar to the German approach, such as Austria or Switzerland. However we believe that they still can be generalized and/or translated to the specific barriers in the cooperation between protagonists in the rehabilitative health care system in other countries.

Recommendations specific to the German health care system include suggestions regarding shortening the rehabilitation report or the use of a standardized communication format in which RPs would communicate the results of their assessment by checking boxes with predefined terms relating to occupational tasks. Two German pilot projects tested similar interventions to improve the communication between stakeholders and found a positive effect. However, both studies were assessed as prone to a high risk of bias (Kuehn et al. 2008; Schwarze et al. 2013). While such tools may be specifically useful for the German rehabilitation system, the barrier of timely transmission of relevant findings and the translation of findings from one specific setting or expert group to another is well known in health services research. Delayed and/or insufficient transfer of information are especially common in the discharge communication between hospital-based to outpatient health care providers (Kripalani et al. 2007a, b; Kattel et al. 2016). It is therefore likely that similar solutions may be useful in the rehabilitation systems in other countries. Although not specifically focused on rehabilitation, the international literature supports structured formats and technology solutions as well as standardized language in order to improve availability, completeness, timeliness, and quality of discharge information from hospitals to out-patient health care providers (Kripalani et al. 2007a, b; Arora et al. 2009; Hesselink et al. 2012; Kattel et al. 2016).

While the suggested interventions regarding finances were identified in a qualitative study on the general cooperation of German OPs and GPs (Mosshammer et al. 2014), they were also reported in two Dutch questionnaire surveys on the role of OPs in the rehabilitation process. In these studies, GPs and RPs suggested increasing remuneration for cooperation with OPs (Buijs et al. 1999; van Amstel and Buijs 2000). Although financial incentives to improve cooperation between protagonists in the health care system may pose a solution, a Cochrane review with a focus on the quality of care provided by primary care physicians found that there is insufficient evidence regarding the effectiveness or non-effectiveness of financial incentives (Scott et al. 2011).

The proposal of introducing or strengthening evaluation and feedback mechanisms is supported by a Cochrane review by Ivers et al. The review assessed the concept that healthcare professionals may be prompted to modify their practice when given performance feedback showing that their clinical practice is inconsistent with a desirable target. This review found moderate evidence that audit and feedback can lead to small but potentially important improvements in professional practice. However, none of the studies included in this review looked explicitly at the topic of this study, and a transferability of the results to the specific circumstances addressed in this study has yet to be assed (Ivers et al. 2012).

Some participants stated that OPs needed to better explain their role, function, and the concept of professional confidentiality to their patients. The need for such clarification is supported by two German publications which indicated that the position and function of the OP often was not clear to employees and employers (Glomm 2001; Dzuck et al. 2002). A qualitative study from the Netherlands concluded that patients viewed the cooperation between OPs and curative physicians from a strategic perspective in which their own interests were the key decisive factors (Plomp et al. 2011). If patients could be convinced that OPs were working in their interest, they could be more supportive of interdisciplinary collaboration.

Studies from Germany, the Netherlands, Belgium and Austria also found that RPs (van Amstel and Buijs 2000; Rijkenberg 2012; Mueller et al. 2013) and GPs (Buijs et al. 1999; Mosshammer et al. 2012) who are unaware of the OP’s functions could pose a barrier to cooperation. A number of studies (i.a. from the Netherlands) have also reported mistrust of OPs among GPs (Buijs et al. 1999; Nauta and von Grumbkow 2001; Pfaff et al. 2009; Mosshammer et al. 2011) and RPs (van Amstel and Buijs 2000; Vroeijenstijn-Nguyen and Brenner 2007) in Germany and the Netherlands regarding, for example, conflicts of interest or adherence to confidentiality regulations. Buijs et al. already concluded in 1999 that OPs must clarify their position to GP colleagues to overcome obstacles to cooperation (Buijs et al. 1999). This is supported by two Dutch surveys among RPs and GPs concluding that if OPs could clarify how they were going to use the patient’s data and that they were working in the interest of patients, this could reduce concerns about cooperation (van Amstel and Buijs 2000; Buijs et al. 2009).

Our participants suggested introducing joint continuing medical education programs to strengthen and facilitate interdisciplinary communication and to build interdisciplinary relationships. Similar proposals have also been made in German qualitative studies on interfaces in the rehabilitation process (Pohontsch and Deck 2011) and cooperation between GPs and OPs in general (Mosshammer et al. 2014). Nauta et al. tested in a Dutch setting whether a joint vocational training program would improve cooperation and trust between junior doctors training to become GPs and OPs. They found that junior GPs’ trust increased after the program and that it helped them to overcome prejudices against OPs. However, this effect concerning the junior GPs’ trust disappeared after 3 months (Nauta et al. 2006). In another Dutch study, a training program did not lead to an increased collaboration between GPs and OPs on lower back pain (Faber et al. 2005). A Cochrane review of the international literature on interprofessional education programs found weak evidence that such programs can have slightly positive effects on cooperation between physicians and other health professionals (Reeves et al. 2013). However, the transferability of the results on collaboration between physicians of different disciplines is unclear. An interest in joint professional training programs was voiced in two surveys by a majority of OPs and RPs from Belgium, Austria, and the Netherlands (Vroeijenstijn-Nguyen and Brenner 2007; Rijkenberg 2012). By contrast, in a qualitative study from Germany a majority of GPs rejected the proposal of joint quality circles with OPs (Mosshammer et al. 2014). The suggested exposure of RPs to the patients’ workplaces has been found to have a strong evidence basis regarding the reduction of work disability duration (Franche et al. 2005).

In a study conducted within the same research project as this study, we assess the role of intergroup dynamics in general as well as the role of negative or stereotypical group perceptions in particular as a barrier to cooperation, based on the Social Identity Approach by Tajfel and Turner (1979, 1986). Based on this theoretical approach, the study identified numerous divergent perceptions (i.e. regarding roles, responsibilities and capabilities) among the specialist groups, as well as negative perceptions, especially about OPs. Both, divergent and negative perceptions are linked to barriers to cooperation. Based on this theory-driven assessment, we propose solutions for resolving conflicts in intergroup dynamics building on approaches, which themselves are based on or are linked to the Social identity approach, i.e. the model to resolve intractable identity-based conflicts (IIC) or the contact hypothesis (Stratil et al. submission in process).

Some of the suggestions proposed by participants seem to indicate a heterogeneous level of knowledge regarding rehabilitation services. For example statements of GPs suggesting an unawareness of existing evaluation schemes or the financing of consolidative post-rehabilitation programs or of one RP admitting to be unaware of the role of OPs in the rehabilitation system. While not explicitly stated by participants, overcoming a lacking knowledge on processes in the rehabilitation system could pose a possible solution. A systematic assessment of informational needs offers a promising field for future research.

Our study has several strengths. We were able to achieve high levels of heterogeneity in our sample of interviewees, e.g. regarding work experience of the different physicians and the disease profile of rehabilitants. Moreover, we included patients as main stakeholders. They made only few suggestions in regards to improving the cooperation, and mostly discussed barriers to cooperation and problems they had experienced during their treatment. As most suggestions proposed by the physicians focused on problems experienced by patients as well, we believe the suggestions to work in their interest. A limitation of the study is that the composition of our focus groups deviated from the composition specified in our protocol (Voelter-Mahlknecht et al. 2017), especially concerning the number of OPs among the participants and their working profile. We believe the perception of different roles of OPs is still represented in our sample, as some OPs had worked as employees of occupational service providers and as staff doctors in the past. As a strong heterogeneity of rehabilitation clinics in regards to OP–RP-cooperation has been indicated by some studies, we cannot exclude the possibility of a bias in the RPs and rehabilitants perception due to unwanted group effects. As participants were aware that the interviews were conducted by occupational health experts, biased responses due to social desirability are possible, but we believe this risk can be considered low due to the richness of our data and the critical statement made in the discussions. We conducted FGDs with homogenous professional groups in order to have participants discuss less constrained and to allow them talk more freely about negative or possibly prejudicial attitudes regarding the other medical specialists or patients. As numerous critical statements were made by participants about other groups of participants, we consider the FGDs with heterogeneous participants of a homogenous professional group to be successful. Inter-professional discussion took place within the validation workshop held in January 2015 where OPs and GPs participated. We will consider conducting FGDs with mixed professional groups based on the finding of this study, if new resources can be acquired.

In this study, we present suggestions to overcome problems and improve collaboration at the interprofessional interfaces of the rehabilitation process in Germany which in part may be transferred to other countries, too. This study builds on an earlier publication that has outlined possible obstacles to cooperation in terms of organizational, interpersonal, and structural barriers. We suggest that stakeholders focus on organizational procedures, education and information interventions, and on the promotion of cooperation, as these interventions may be implemented by the stakeholders themselves in their everyday working routine. Changing aspects of finance and regulation may be more effective although more complicated to establish and therefore more suitable as long term solutions.

Based on the literature, the qualitative study on barriers to cooperation and the results of this qualitative study on possible solutions, we believe a key aspect lies in changing the perception of and knowledge about the role and function of OPs in general and in the rehabilitation process in particular.

## Electronic supplementary material

Below is the link to the electronic supplementary material.

Supplementary material 1 (PDF 346 kb)
