# Supplementary material for: Optimizing cooperation between general practitioners, occupational health and rehabilitation physicians in Germany: a qualitative study
Source: Int Arch Occup Environ Health. 2017 Jul 5;90(8):809–21. doi: 10.1007/s00420-017-1239-6 (PMC5640724; doi:10.1007/s00420-017-1239-6)

Category system used for coding the transcripts

This figure describes the category system with the main categories, categories and subcategories.

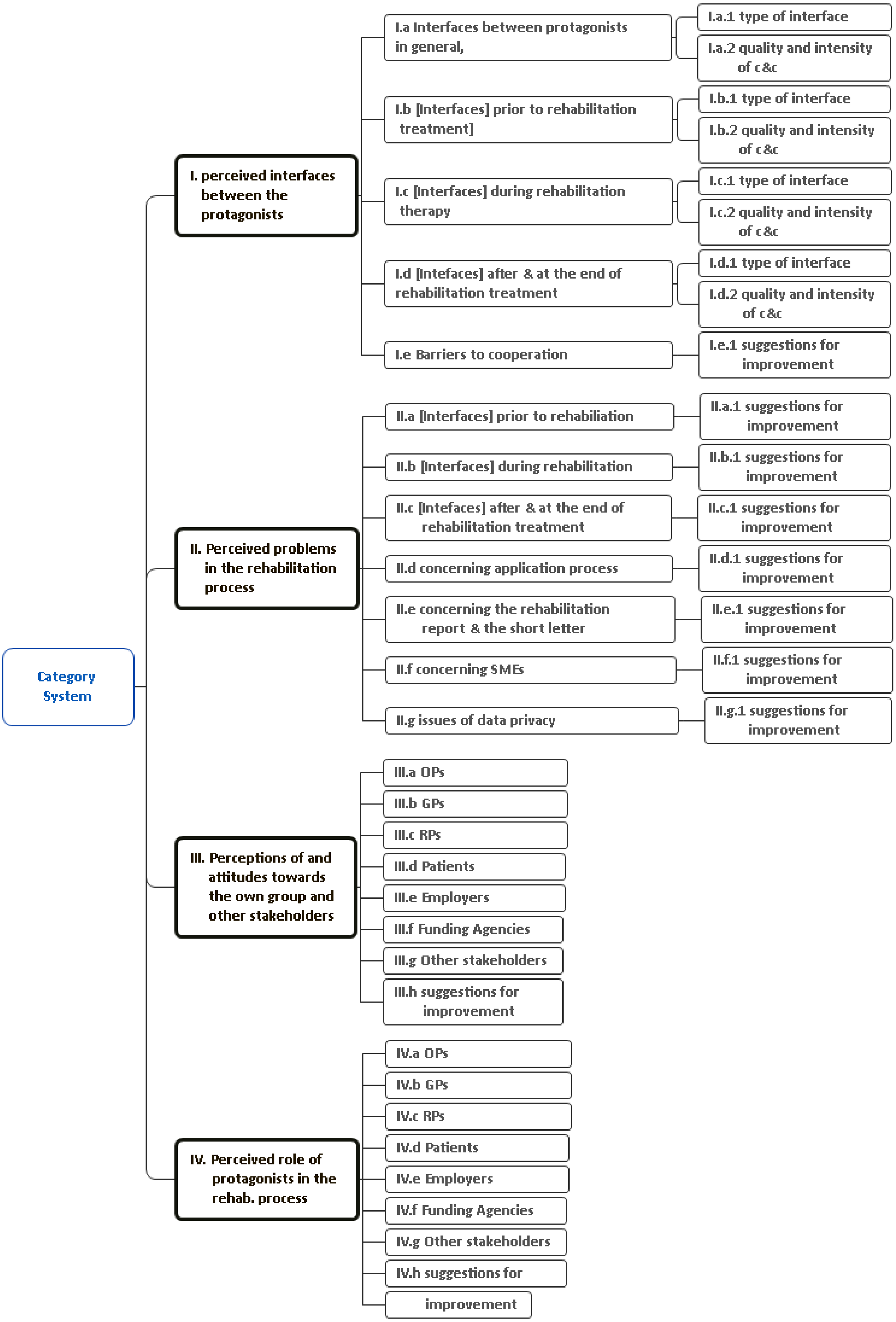

Supplement: Supplementary file 1 — Supplementary material 1 (PDF 346 kb) [file 420_2017_1239_MOESM1_ESM.pdf]
